# Primary Pulmonary Involvement in Mucosa-associated Lymphoid Tissue Lymphoma

**DOI:** 10.7759/cureus.5110

**Published:** 2019-07-09

**Authors:** Khushali Jhaveri, Derek J Dimas, Abhay Vakil, Salim Surani

**Affiliations:** 1 Internal Medicine, Medstar Washington Hospital Center, Washington, USA; 2 Internal Medicine, Christus Spohn Hospital Corpus Christi, Corpus Christi, USA; 3 Internal Medicine, University of North Texas, Denton, USA; 4 Internal Medicine, Texas A&M Health Science Center, Temple, USA

**Keywords:** pulmonary nodule, lung cancer, lymphoma, non-hodgkin’s lymphoma, lymphoproliferative disorders, malt lymphoma

## Abstract

Pulmonary nodules have a broad differential diagnosis with primary lung cancer, lung metastases, benign tumors, carcinoid tumors, and infectious granulomas as their common cause. While relatively rare, pulmonary lymphoproliferative disorders such as primary pulmonary lymphomas, primary pulmonary plasmacytomas, secondary lymphomas involving the lung, multiple myeloma involving the lung, leukemias involving the lung should be considered in these patients presenting with lung nodules. Primary pulmonary non-Hodgkin’s lymphoma (NHL) is an extremely rare lung tumor accounting for 0.4% of all lymphomas. Mucosa-associated lymphoid tissue (MALT) lymphoma accounts for about 70%-90% of all primary pulmonary lymphomas, constituting less than 0.5% of all the lung neoplasms. Though it usually remains localized, it is a clonal B-cell neoplasm with a potential for systematic spread and transformation to an aggressive B-cell lymphoma. We hereby discuss the case of a 66-year-old woman with primary pulmonary MALT lymphoma.

## Introduction

Pulmonary nodules commonly seen on imaging have a broad differential diagnosis. Pulmonary lymphoproliferative disorders should be considered in the differential diagnosis of these patients presenting with lung nodules. Primary pulmonary non-Hodgkin’s lymphoma (NHL) is an extremely rare lung tumor accounting for 0.4% of all lymphomas [1]. Mucosa-associated lymphoid tissue (MALT) lymphoma accounts for about 70%-90% of all primary pulmonary lymphomas [2], constituting less than 0.5 % of all the lung neoplasms. We hereby discuss the case of a 66-year-old woman with primary pulmonary MALT lymphoma.

## Case presentation

A 66-year-old non-smoker woman presented to the emergency department with worsening of maculopapular skin rash of six weeks. The rash had gradually progressed to involve all the extremities with generalized pruritus and failure to resolve after a trial of over the counter antihistamines. She also reported loss of appetite with 10 pounds weight loss in the last one month without any associated cough, fever, hemoptysis, dyspnea or joint pain. She denied any allergies, recent sick contacts, insect bites, skin rash in the past and failed to recall any new medication or dietary supplement use. She worked as an accountant and had no illicit drug use or risk factors for immunodeficiency virus (HIV).

On physical examination, the patient was thin but not cachectic. Vital signs on examination were blood pressure of 112/84 mmHg, pulse of 61/min, respiratory rate of 12/min, temperature of 97○F, and saturation on room air of 98%. She was alert and awake with a normal mentation. There was no palpable lymphadenopathy. Respiratory exam revealed bilaterally equal breath sounds with no wheezing or rales. Cardiac and abdominal examinations were normal. She had non-blanching maculopapular rash involving all the extremities with a positive Darier’s sign. There was no rash on her chest or abdominal wall. She had normal strength and sensations in all extremities.

**Figure 1:**
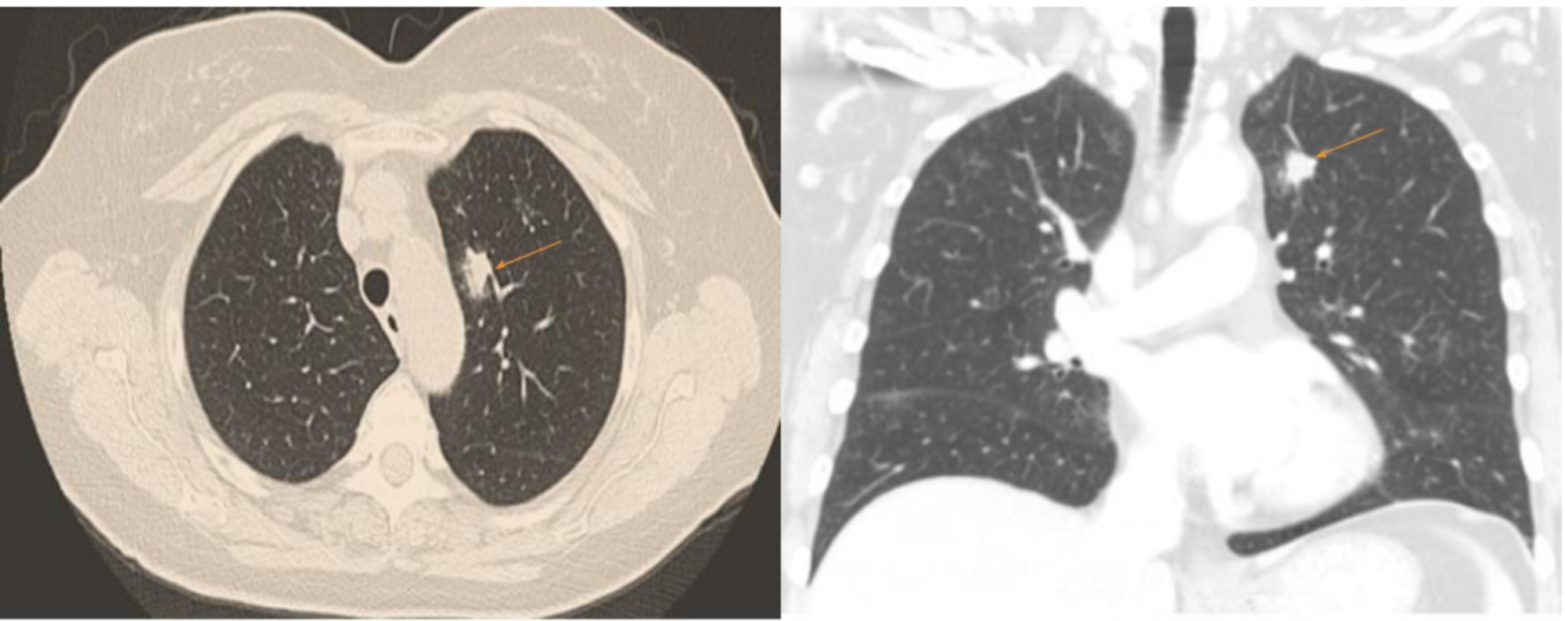
Computed tomography (CT) of the chest showing 2.1 x 0.9 cm left upper lobe nodule

Initial laboratory results included a normal complete blood count (CBC). Renal function, electrolytes, and liver function tests were normal. Protein and albumin were 9.5 g/dL and 3.9 g/dL respectively. Serum protein electrophoresis showed immunoglobulin M (IgM) spike with a level of 2272 mg/dL (normal 37-286 mg/dL). Her serum tryptase level was elevated at 32 ng/mL (normal1-11.4 ng/mL). Skin biopsy confirmed the diagnosis of mastocytosis. Bone marrow examination was followed by computed tomography (CT) scan of the chest, abdomen, and pelvis to evaluate systemic involvement in mastocytosis and assess associated hematologic and solid organ malignancies. CT scan of the chest showed 2.1 x 0.9 cm left upper lobe nodule and multiple other ground glass nodules without any lymphadenopathy (Figure 1).

**Figure 2:**
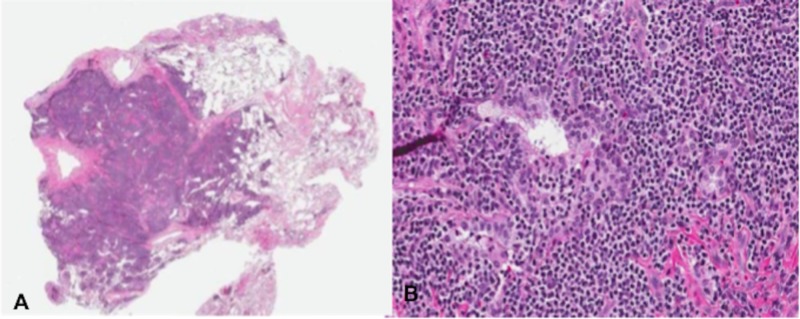
A) Low and B) high power showing nodular lymphoid aggregates with lymphoepithelial lesions

Abdominal CT did not show any organomegaly or lymphadenopathy. Bone marrow biopsy failed to show any significant abnormality. Elevated fluorodeoxyglucose (FDG) uptake and nodules which increased in size and density over six months led to a video-assisted thoracoscopic wedge resection of the largest solid lung nodule. Histopathologic examination of the resected tissue revealed nodular lymphoid aggregates with lymphoepithelial lesions (Figure 2).

**Figure 3:**
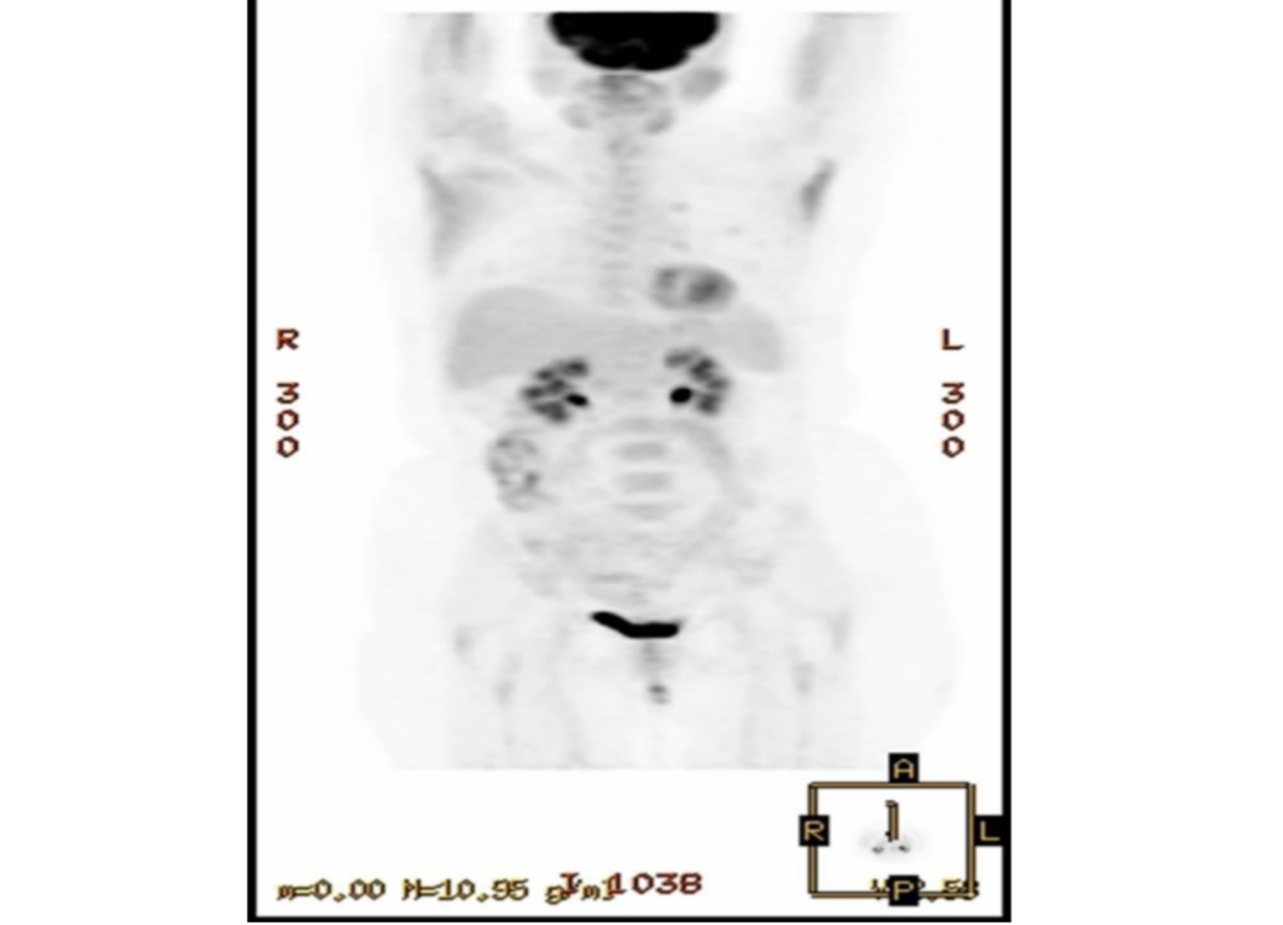
Positron emission tomography (PET) scan showing no hypermetabolic lesion

Immunohistochemical staining showed infiltration by cluster of differentiation (CD)20 positive B cells with aberrant CD43 expression. The analysis demonstrated the cells to be negative for CD5, CD10, and BCL-6 indicating a MALT lymphoma. The patient was diagnosed with primary pulmonary involvement in extra-nodal marginal zone lymphoma of MALT origin (MALT lymphoma). Our patient’s skin rash and pruritus resolved spontaneously over the course of the next six weeks. Positron emission tomography (PET) scan failed to identify any other hypermetabolic lesion (Figure 3).

Repeat CT chest at six and 12 months after resection failed to show any evidence of recurrence or metastasis and the patient continued to be symptom free.

## Discussion

Pulmonary lymphoproliferative disorders are a group of localized or disuse processes involving the pulmonary and pleural lymphatic system. These disorders include primary pulmonary lymphomas, primary pulmonary plasmacytomas, secondary lymphomas involving the lung, multiple myeloma involving the lungorleukemias involving the lung. Primary pulmonary lymphoma can affect one or both lungs (parenchyma, small or large airways, or any combination) without any evident extrapulmonary involvement. Primary pulmonary NHL is an extremely rare lung tumor accounting for 0.4% of all lymphomas. MALT lymphoma accounts for about 70%-90% of all primary pulmonary lymphomas, constituting less than 0.5% of all the lung neoplasms [3].

Most of the patients with pulmonary MALT lymphomas are asymptomatic at presentation [4]. However, symptomatic cases present with predominant pulmonary symptoms like cough, dyspnea and rarely hemoptysis [5]. Pulmonary MALT lymphoma is known to be associated with paraproteinemias and mast cell stimulation leading to their abnormal deposition in extra pulmonary tissue [6]. Patients can also present with extrapulmonary symptoms secondary to paraproteinemias and mastocytosis. Our patient had absent pulmonary symptoms but presented with worsening of maculopapular skin rash secondary to mastocytosis. Her serum protein electrophoresis showed IgM spike with a level of 2272 mg/dl (normal 37-286 mg/dL). 

It is believed that pulmonary MALT is not a normal constituent of the lung and usually develops after long-term antigenic stimulation or secondary to chronic inflammation due to infectious and or autoimmune processes [7-8]. It has been suggested that patients diagnosed with pulmonary MALT lymphoma should be evaluated for the presence of underlying autoimmune disorders and or infectious processes [9-10]. MALT lymphoma is also associated with localized deposits of amyloid within the tumor [11-12]. However, MALT lymphoma is not known to be associated with systemic amyloidosis and screening of such patients for systemic amyloidosis is not recommended.

Histopathologic examination of the lung tissue along with immunohistochemical staining is required to confirm the diagnosis of pulmonary MALT lymphoma. No specific immunohistochemical marker has been identified for MALT lymphoma. However, the neoplastic cells in pulmonary MALT lymphoma are usually positive for BCL-2, BCL-10, CD20 and CD43 [7]. Negativity of the lymphoma cells for other markers like IgD, CD5, CD10, BCL-6, and cyclin D1 helps to exclude other B cell lymphomas [7]. In our patient, immunohistochemical staining showed infiltration by CD20 positive B cells and aberrant CD43 expression with cells being negative for CD5, CD10, and BCL-6 indicating a MALT lymphoma.

MALT lymphoma of the lung is usually diagnosed in patients in their late sixties and with a tendency to remain localized. However, it may spread to involve the lymph nodes, stomach or salivary glands. It is associated with a good prognosis with an 84%-94% five-year survival rate and a 72% 10-year survival rate. Due to the rarity of the disease, there is no evidence-based therapeutic strategy based on randomized clinical trials. For localized disease, surgical resection has been the recommended treatment of choice. For advanced disease and for unresectable cases, systemic lymphoma therapy is often recommended, including rituximab. Cases of pulmonary MALT lymphoma responding to long-term clarithromycin therapy have been reported [13-14]. MALT lymphoma may also progress to diffuse large B-cell lymphoma [15].

## Conclusions

Pulmonary lymphoproliferative disorders should be considered in the differential diagnosis of the patients presenting with lung nodules. Pulmonary MALT lymphoma is an extremely rare lung tumor accounting for less than 0.5% of all the lung neoplasms and is known to be associated with paraproteinemias, mastocytosis, and localized amyloid deposition. Histopathologic examination with immunohistochemical staining is necessary to confirm the diagnosis of pulmonary MALT lymphoma. Surgical resection is the recommended treatment of choice in localized disease with an 84%-94% five-year survival rate.
